# Predicting Survival Duration With MRI Radiomics of Brain Metastases From Non-small Cell Lung Cancer

**DOI:** 10.3389/fonc.2021.621088

**Published:** 2021-03-05

**Authors:** Bihong T. Chen, Taihao Jin, Ningrong Ye, Isa Mambetsariev, Tao Wang, Chi Wah Wong, Zikuan Chen, Russell C. Rockne, Rivka R. Colen, Andrei I. Holodny, Sagus Sampath, Ravi Salgia

**Affiliations:** ^1^Department of Diagnostic Radiology, City of Hope National Medical Center, Duarte, CA, United States; ^2^Department of Medical Oncology and Therapeutics Research, City of Hope Comprehensive Cancer Center and Beckman Research Institute, Duarte, CA, United States; ^3^Departments of Interventional Radiology, Nanjing First Hospital, Nanjing Medical University, Nanjing, China; ^4^Applied AI and Data Science, City of Hope National Medical Center, Duarte, CA, United States; ^5^Division of Mathematical Oncology, City of Hope National Medical Center, Duarte, CA, United States; ^6^Department of Radiology, Hillman Cancer Center, University of Pittsburgh Medical Center, Pittsburgh, PA, United States; ^7^Department of Radiology, Memorial Sloan-Kettering Cancer Center, New York, NY, United States; ^8^Department of Radiation Oncology, City of Hope National Medical Center, Duarte, CA, United States

**Keywords:** radiomics, machine learning, survival, lung cancer, brain metastases, brain MRI, artificial intelligence

## Abstract

**Background:** Brain metastases are associated with poor survival. Molecular genetic testing informs on targeted therapy and survival. The purpose of this study was to perform a MR imaging-based radiomic analysis of brain metastases from non-small cell lung cancer (NSCLC) to identify radiomic features that were important for predicting survival duration.

**Methods:** We retrospectively identified our study cohort via an institutional database search for patients with brain metastases from EGFR, ALK, and/or KRAS mutation-positive NSCLC. We segmented the brain metastatic tumors on the brain MR images, extracted radiomic features, constructed radiomic scores from significant radiomic features based on multivariate Cox regression analysis (*p* < 0.05), and built predictive models for survival duration.

**Result:** Of the 110 patients in the cohort (mean age 57.51 ± 12.32 years; range: 22–85 years, M:F = 37:73), 75, 26, and 15 had NSCLC with EGFR, ALK, and KRAS mutations, respectively. Predictive modeling of survival duration using both clinical and radiomic features yielded areas under the receiver operative characteristic curve of 0.977, 0.905, and 0.947 for the EGFR, ALK, and KRAS mutation-positive groups, respectively. Radiomic scores enabled the separation of each mutation-positive group into two subgroups with significantly different survival durations, i.e., shorter vs. longer duration when comparing to the median survival duration of the group.

**Conclusion:** Our data supports the use of radiomic scores, based on MR imaging of brain metastases from NSCLC, as non-invasive biomarkers for survival duration. Future research with a larger sample size and external cohorts is needed to validate our results.

## Introduction

Lung cancer is the second most commonly diagnosed cancer (1). Non-small cell lung cancer (NSCLC) makes up ~85–90% of all lung cancer cases, and 30–50% of patients with NSCLC develop brain metastases (2, 3). Despite advancements in treatment, the survival duration of patients with lung cancer brain metastases remains short, with a poor median survival of 4–8 months after diagnosis (4). Molecular characteristics help to determine whether patients with cancer will respond to targeted therapies thus prolong survival (5). The molecular testing of lung cancer usually screens for genes encoding epidermal growth factor receptor (EGFR), anaplastic lymphoma kinase (ALK) and Kirsten rat sarcoma viral oncogene homolog (KRAS) (6–8). Molecularly targeted medications that can penetrate the central nervous system have improved outcomes in patients with brain metastases from lung cancers with actionable mutations. For example, tyrosine kinase inhibitors, such as erlotinib, have been effective in treating brain metastases in NSCLC patients with EGFR mutations (9). Therefore, the knowledge of molecular mutation status is essential for planning individualized treatments and for predicting survival.

Pathological tissue confirmation and molecular characterization of brain metastases through invasive biopsy or surgical resection are not always possible or practical. In contrast, neuroimaging methods, such as brain magnetic resonance imaging (MRI), are commonly used to non-invasively assess the entire brain to diagnose and to plan treatments for patients with brain metastases. In addition, brain metastases may present with various imaging features depending on the mutation status of the primary NSCLC (10). However, little is known about the relationship between the neuroimaging features of brain metastases and the NSCLC mutation subtypes for survival prediction. There is an unmet need to identify non-invasive neuroimaging biomarkers to predict survival duration for NSCLC patients with brain metastases who may have one of the three most common mutations, i.e., EGFR, ALK, or KRAS.

Radiomics is a computerized method to extract high-dimensional data from non-invasive standard-of-care medical images (11). It can provide a detailed characterization of tumors, in terms of tumor heterogeneity in relation to aggressiveness, which are not perceptible to the human eye (12, 13). In addition, linking imaging features with molecular and immune characteristics will contribute valuable information that is critical for cancer treatment and prognosis (14). Furthermore, the radiomic approach allows the non-invasive analysis of treatment response and prognosis at multiple time points, which is not feasible or practical using invasive biopsies. Radiomic scores, which incorporate information about key imaging features, have shown potential as biomarkers for predicting survival in patients with lung cancer and breast cancers (13, 15, 16). However, to the best of our knowledge, no published studies have used radiomic analysis of brain metastases to predict survival duration of patients with NSCLC according to their mutation status.

Here, we performed a MRI radiomic analysis of brain metastases for survival duration in patients with NSCLC. We hypothesize that MRI radiomics of brain metastases could be used to predict survival duration in patients with NSCLC. Our objective was to use radiomic features extracted from MR images of the brain metastases to build machine learning models for predicting survival durations of patients with NSCLC according to the specific mutation status of their primary NSCLC, i.e., EGFR, ALK, or KRAS. In addition, we constructed a radiomic score for each mutation-positive group to predict whether the patients survived longer or shorter than the median survival duration for each group.

## Methods

### Patient Selection and Imaging Acquisition

We retrospectively identified consecutive patients for this study by searching the Thoracic Oncology Registry for all lung cancer patients treated at City of Hope National Medical Center (Duarte, CA, USA) between 2009 and 2017. Eligibility criteria included the following: diagnosis of NSCLC; confirmation via genotype testing of an EGFR, ALK, and/or KRAS mutation in the primary NSCLC tumors; and having brain MRI scans performed to diagnose brain metastases but before initiating treatment for the brain metastases. Patient demographic data, survival information including date of death or last follow-up, and mutation status were abstracted from electronic medical records (Table 1). The Institutional Review Board at City of Hope National Medical Center approved this study and waived informed consent due to its retrospective nature. The study was conducted in accordance with the Declaration of Helsinki.

**Table 1:** Demographic information for the study cohort.

	***EGFR* (+)**	***ALK* (+)**	***KRAS* (+)**	***p*-value**
	***N* = 75**	***N* = 21**	***N* = 15**	
**Age**				
Mean ± SD	57.43 ± 12.09	53.81 ± 14.79	63.67 ± 6.40	0.09
**Race**				
Caucasian	34 (45.33%)	13(61.90%)	11 (73.33%)	
Asian	35 (46.67%)	7 (33.33%)	1 (6.67%)	0.016
Other^1^	6 (8%)	1 (0.04%)	3 (20%)	
**Gender**				
Male	24 (32%)	8 (38.10%)	5 (33.33%)	0.83
Female	51 (68%)	13 (61.90%)	10 (66.67%)	
**History of Smoking**				
Yes	20 (26.67%)	5 (23.80%)	12 (80%)	<0.001
No	55 (73.33%)	16 (76.19%)	3 (20%)	

1 *American Indian or Alaska Native, African American, Native Hawaiian, or Pacific Islander*.

*EGFR, epidermal growth factor receptor; ALK, anaplastic lymphoma kinase; KRAS, Kirsten rat sarcoma viral oncogene homolog*.

Brain MR images including both the T1-weighted contrast-enhanced (T1C) and T2-weighted fluid-attenuated inversion recovery (FLAIR) sequences were retrieved from our Picture Archiving and Communication System. Brain MR scans were obtained from the same in-house 3T VERIO Siemens scanner (Siemens, Erlangen, Germany). T1C sequence was acquired with axial T1-weighted three-dimensional (3D) magnetization prepared rapid gradient echo (MPRAGE) imaging after intravenous administration of MultiHance® (gadobenate dimeglumine) at 0.1 mmol/Kg. The FLAIR sequence for the peritumoral edema was acquired with routine imaging protocol. Detailed scanning parameters have been reported in our previous study (10).

### Brain Tumor Segmentation

For image segmentation, we co-registered T1C and FLAIR images into the same geometric space under an affine transformation as established by the elastix toolbox (17). We segmented the T1C and FLAIR images for enhancing tumor and peritumoral edema, respectively. We performed image transformation and re-slicing with FSL scripts (https://fsl.fmrib.ox.ac.uk/fsl/fslwiki/).

**Figure 1:**
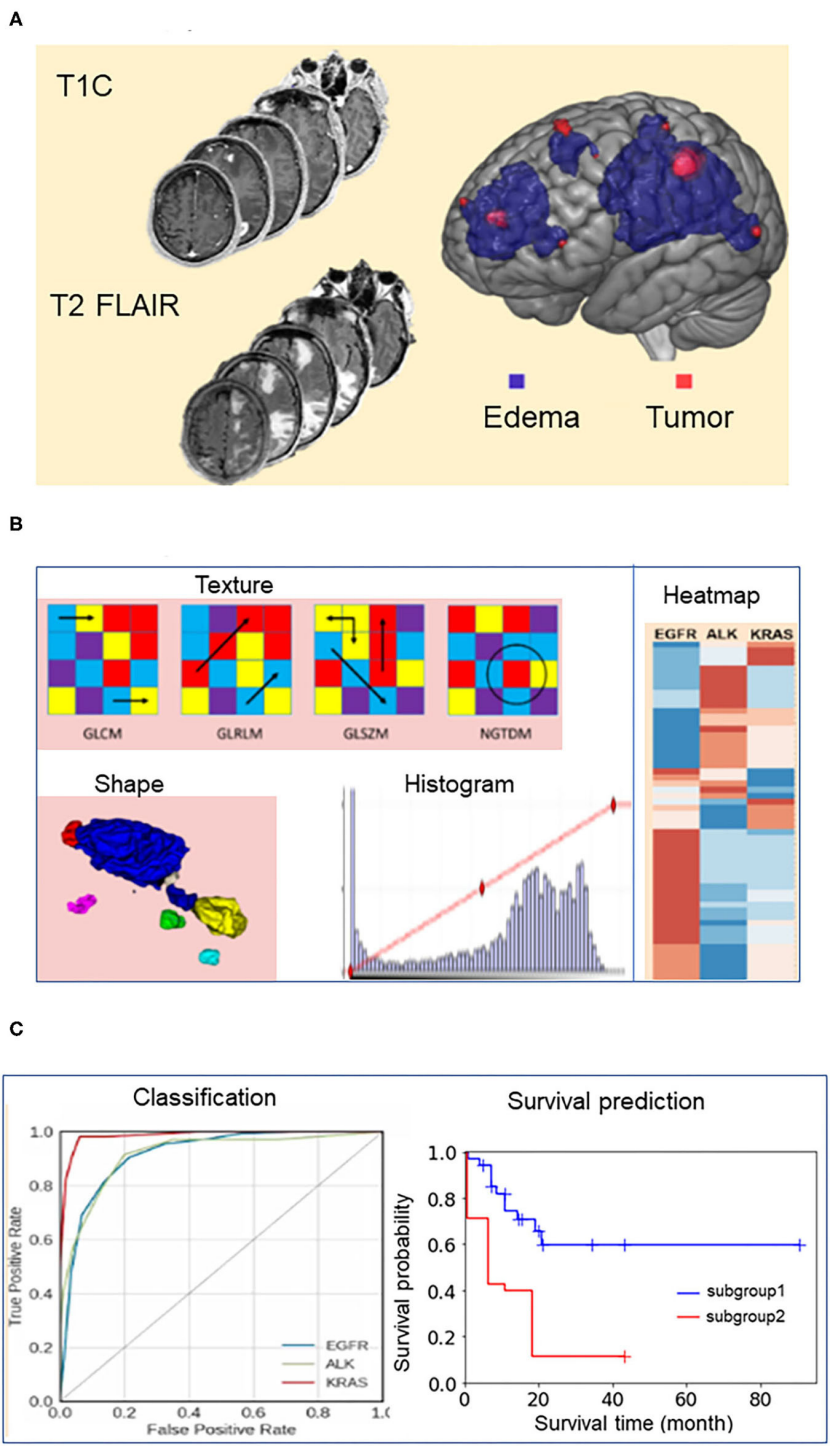
Schema for brain tumor segmentation, radiomic feature extraction, and predictive modeling. **(A)** Representative tumor segmentation images from post-contrast T1-weighted (T1C) and T2-weighted fluid-attenuated inversion recovery (FLAIR) data. **(B)** Illustrations of radiomic features extracted from the brain tumor images, including texture, shape, and intensity. GLCM, Gray Level Co-occurrence Matrix; GLRLM, Gray Level Run Length Matrix; GLSZM, Gray Level Size Zone Matrix; NGTDM, Neighboring Gray Tone Difference Matrix. **(C)** Receiver operating characteristic (ROC) curves for the models predicting the survival durations of patients in each of the three mutation-positive groups (EGFR, ALK, and KRAS mutation-positive groups) and representative survival duration analysis.

Subsequently, we used ITK-SNAP, an open-source 3D image analysis software (www.itksnap.org) to contour the tumor boundaries of both T1C (for the enhancing tumor) and FLAIR (for the peritumoral edema) images in a semi-automated fashion on a slice-by-slice basis (18). This semi-automated method consisted of the two steps. First, the ITK-SNAP software automatically placed a region of interest box around the tumors. Second, the tumor boundaries were manually drawn slice-by-slice by our trained research personnel (NY, TW, and BC). One researcher (NY) was a neuroimaging researcher with 2 years of experience in tracing tumors for radiomic research. The other two researchers (TW and BC) were neuroradiologists with a combined 20 years of experience in neuroimaging. Discrepancy during tumor segmentation was resolved by consensus of the research group. We have reported the details of brain tumor segmentation previously (10). The imaging delineation (mask) of the two segmented phenotypes (enhancing tumor and peritumoral edema) were exported for radiomic analysis. Our analysis included up to 10 of the largest tumors from each patient, limited to tumors >5 mm in diameter because smaller tumors could not be reliably segmented for 3D analysis. Our dataset consisted of 452 lesions from 110 patients. Figure 1 presents the schema for brain tumor segmentation, radiomic feature extraction, and predictive modeling for survival duration.

To assess the consistency of image segmentation and the stability of radiomic features extracted for modeling, two researchers (NY and TW) independently performed tumor segmentation on the brain images from 20 randomly selected patients with the results being blinded to each other. We then used their segmentation results to test the inter-observer variability. In addition, one researcher (NY) repeated the brain tumor segmentation twice with 1 month apart for testing the intra-observer variability. We used the interclass correlation coefficient (ICC) test to assess the consistency of the radiomic features for both inter-observer and intra-observer variability. An inter-observer and intra-observer ICC > 0.80 was considered stable for tumor segmentation and radiomic feature extraction. The inter-observer ICC between the two researchers (NY and TW) for tumor segmentation achieved at 0.96 ± 0.04 in a range from 0.87 to 0.99 and for edema segmentation achieved at 0.95 ± 0.05 in a range from 0.80 to 0.99. The intra-observer ICC between the two measurements by the same researcher (NY) achieved 0.99 ± 0.006 (range from 0.97 to 1.00), and 0.99 ± 0.007 (range from 0.97 to 1.00) for segmentation of tumor and edema, respectively. The results indicated favorable inter- and intra-observer reproducibility and stability for tumor segmentation and subsequent radiomic feature extraction.

### Radiomic Feature Extraction and Selection

The image preprocessing and radiomic feature extraction have been previously reported by our group (10). Briefly, we preprocessed each of the T1C or FLAIR images using a pipeline consisting of three steps: (i) skull-stripping using the Brain Extraction Tool (BET; http://fsl.fmrib.ox.ac.uk/fsl/fslwiki/BET) and Free Surfer (https://surfer.nmr.mgh.harvard.edu/); (ii) bias field correction using the routine N4ITKBiasFieldCorrection of nipype (https://nipype.readthedocs.io/en/0.12.0/users/index.html); (iii) image intensity normalization using an algorithm to standardize the intensity scales across MR images of the same contrast (19). Subsequently, we applied six different filters (Wavelet, Laplacian of Gaussian, Square, Square Root, Logarithm, or Exponential) to each of the preprocessed images, generating six derived images. Therefore, there were 12 derived images associated with each brain lesion, 6 for each of the two original (T1C and FLAIR) images. Finally, we performed radiomic feature extraction using an open-source python package PyRadiomics (https://pyradiomics.readthedocs.io/en/latest/) (20) on each derived image by applying a tumor or edema mask based on the modality of the original image, i.e., applying the tumor mask on the six images derived from the original T1C image, and applying the edema mask on the six images derived from the original FLAIR image. We extracted three types of radiomic features from each image including: (i) textural features, including Gray Level Co-occurrence Matrix (GLCM), Gray Level Run Length Matrix (GLRLM), Gray Level Size Zone Matrix (GLSZM), Neighboring Gray Tone Difference Matrix (NGTDM), and Gray Level Dependence Matrix (GLDM); (ii) shape-based features, including Volume, Surface Area, and Sphericity; and (iii) intensity-based features, such as Minimum, Maximum, and Mean. We extracted a total of 2,786 radiomic features from the 12 derived images for each lesion.

We performed feature selection in two steps. First, we selected 2,520 stable features from the total of 2,786 features based on the inter-observer ICC test with a threshold of 0.8 (corrected *p* < 0.05). Second, from those 2,520, the 50 most relevant features for model building were selected using a minimum redundancy and maximum relevance (MRMR) algorithm (21).

### Building Predictive Models for Survival Duration

We dichotomized the patients in each mutation-positive group into two subgroups, i.e., shorter and longer survival subgroups, by assigning the patients with survival duration shorter than the median of the mutation-positive group to the shorter survival subgroup and the remaining patients to the longer survival subgroup. Subsequently we built independent machine learning models for each mutation-positive group to predict whether a patient survived longer than the median survival duration of the group. We evaluated the predictive performance of the machine learning models through leave one out cross validation (LOOCV) using four commonly used performance metrics including the area under the curve (AUC) of the receiver operating characteristic curves (ROC), the specificity, sensitivity and the prediction accuracy (22). We used an open source software scikit-learn for the machine learning model training and evaluation (23). Model training and prediction were tumor-based rather than patient-based, meaning each tumor was treated as an independent instance. The synthetic minority over-sampling technique (SMOTE) was used to improve learning using imbalanced datasets (24).

We built the predictive models using the 50 radiomic features alone or together with 18 additional features including demographic, clinical, and tumor information. Demographic information included gender (male, female), race (Caucasian, Asian, and other), and smoking history (yes, no). Clinical information included the presence or absence of extracranial metastases at 11 sites (bone, lymph, liver, lung, kidney, pancreas, breast, spinal cord, mediastinum, pericardium, and pleura). Tumor information included the number of tumors, the volume of the enhancing tumor core, and the edema/tumor volume ratio. The MRMR-based feature selection was performed in each round of LOOCV process, i.e., 50 most relevant radiomic features were selected using the MRMR algorithm using the training dataset (sample size equals *N*−1 for a *N* sample dataset) after leaving one sample out as the test dataset.

### Selection of Machine Learning Algorithm

We used the gradient boosting classifier to build the machine learning models for predicting the survival durations of all three mutation groups. We selected this algorithm using a model selection process that has been previously described (10). Briefly, (a) we tested 30 classifiers implemented in Scikit-Learn software (23) and evaluated their performance using leave-one-out cross validation (LOOCV), (b) we subsequently ranked their performances according to the area under the curve (AUC) of the receiver operating characteristic curve (ROC) of each model, and (c) we selected the algorithm, Gradient boosting classifier, because it was the only one ranked among top three algorithms for modeling each of the three patient groups.

Table 2 presents the performance data for the top three algorithms for each of the three mutation groups. The performance metrics include accuracy, AUC, sensitivity, and specificity. A total of five classifiers (ada boosting, random forest, extra tree, bagging, and gradient boosting) ranked among the top three classifiers for modeling at least one of the three mutation groups. Gradient boosting classifier was the only classifier ranked among top three for all three mutation-positive groups, therefore, we used this algorithm to build the predictive models for all three mutation groups.

**Table 2:** Performance metrics for the top three machine learning algorithms for predicting whether patients survive longer than the group median in the EGFR, ALK, and KRAS mutation-positive groups using radiomic features only.

**Mutation**	**Classifier**	**Accuracy**	AUC^*^	**Sensitivity**	**Specificity**
EGFR	Ada Boost Classifier	84.30%	0.905	86.00%	82.00%
	Bagging Classifier	84.00%	0.915	90.00%	79.00%
	Gradient Boosting Classifier	88.10%	0.95	90.00%	87.00%
ALK	Gradient Boosting Classifier	85.70%	0.92	88.00%	83.00%
	Random Forest Classifier	77.80%	0.93	95.00%	68.00%
	Extra Trees Classifier	85.70%	0.936	90.00%	81.00%
KRAS	Extra Trees Classifier	78.70%	0.913	84.00%	75.00%
	Gradient Boosting Classifier	85.10%	0.955	83.00%	87.00%
	Ada Boost Classifier	95.70%	0.957	100.00%	92.00%

* *AUC, area under the receiver operating characteristic curve. EGFR, epidermal growth factor receptor; ALK, anaplastic lymphoma kinase; KRAS, Kirsten rat sarcoma viral oncogene homolog*.

### Statistical Analysis and Radiomic Score

#### Demographic Data

We used analysis of variance (ANOVA) to determine the statistical significance of group differences in age. The normality of the distribution was tested using the Shapiro-Wilk test, and the homoscedasticity (the three groups have equal variance) was tested using Bartlett's test implemented in SciPy. We used Fisher's exact test to determine the statistical significance of group differences in the distributions of the categorical variables, including gender, race, smoking history, histology, and other metastatic sites. *P* < 0.05 were considered statistically significant. We used the statistical analysis package in the SciPy: open source scientific tools for Python library (https://www.scipy.org/) for the analysis described above.

#### Survival Analysis and Radiomic Score

**Figure 2:**
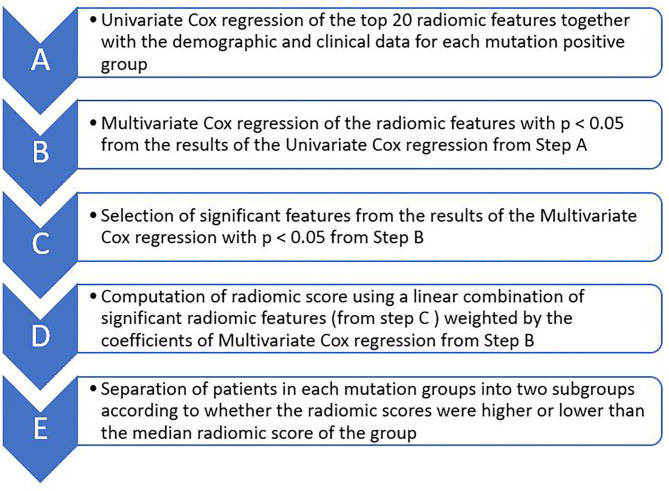
Major steps of Cox proportional hazard regression analysis for determining the effects of radiomic features on survival durations of patients for each of the three mutation-positive group (EGFR, ALK, and KRAS mutation-positive groups). The top 20 radiomic features (step A) were selected based on the feature importance as determined by the multivariate Cox regression during classifier training.

We selected radiomic and clinical features that were important for patients' survival duration and subsequently computed radiomic score for each patient by sequentially performing univariate and multivariate Cox proportional hazard regression through the following steps (Figure 2): **(A)** Selecting 20 radiomic features potentially associated with patients' survival duration. In this step, we computed the feature importance of the 50 radiomic features used in the machine learning models using scikit learn software as described in the Section: Building Predictive Models for Survival Duration) and selected the top 20 radiomic features according to the feature importance value (Supplementary Table 1, Supplementary Material); **(B)** Performing univariate Cox regression using each of the selected top 20 radiomic features (one by one) and selected those with *p* < 0.05 in the analysis; **(C)** Performing multivariate Cox regression using the above selected radiomic features together with the 18 clinical feature (described in Section Building Predictive Models for Survival Duration) and chose those with *p* < 0.05 in the analysis as the final selected radiomic and clinical features; **(D)** Computing radiomic score for each patient in each mutation-positive group using a linear combination of the features selected in step C weighted by the coefficients determined by the multivariate Cox regression. We divided each mutation group into two subgroups according to the radiomic scores. In each mutation group, those patients with higher radiomic scores than the group median were assigned into the high radiomic score subgroup, and the rest of the patients in the mutation-positive group were assigned into the subgroup with lower radiomic score. We tested the statistical significance of the differences in the median survival durations between the two subgroups in each mutation-positive group using log rank test. We used log rank test to compare the median survival durations of patients in the EGFR, ALK, and KRAS mutation-positive groups. We used Lifelines, an open source software in Python (https://lifelines.readthedocs.io/en/latest/), for the survival analysis and presentation described in this section.

## Results

### Patient Information

The 110 patients in this study cohort [mean age: 57.51 ± 12.32 years (range: 22 to 85 years), M:F = 37:73] were separated into three groups according to mutation status of the three oncogenes EGFR, ALK, and KRAS. In this cohort, 75 patients had EGFR mutation, 21 had ALK mutation, and 15 had KRAS mutation in their primary NSCLC, respectively (Table 1). There was one patient who was positive for both ALK and EGFR mutations. A detailed summary of the demographic and clinical information for the cohort has been reported previously focusing on classification of mutation status from lung cancer brain metastases (10). Briefly, there were statistically significant group differences for the two categorical variables, race (*p* < 0.05) and smoking history (*p* < 0.001). There was a significant difference in the racial distribution of the EGFR and KRAS groups (*p* = 0.005), and the KRAS group had a higher percentage of smokers than the EGFR (*p* = 0.0002) and ALK (*p* = 0.0036) groups.

We also compared the demographic data between the mutation-positive group and the mutation-negative groups for each gene mutation, i.e., EGFR (+) vs. EGFR (–), ALK (+) vs. ALK (–), and KRAS (+) vs. KRAS (–). There was a significantly greater percentage of Asian patients in the EGFR (+) group than the EGFR (–) group (*p* = 0.042). The KRAS (+) group was significantly older than the KRAS (–) group (*p* = 0.002). There was a higher percentage of smokers in the KRAS (+) group than the KRAS (–) group (*p* = 0.0001).

The median survival durations for EGFR, ALK, and KRAS mutation-positive groups were 12.7, 20.9, and 17.0 months, respectively. The pair-wise log-rank test indicated that the median survival duration of the ALK mutation-positive group was significantly longer than that of the EGFR mutation-positive group (*p* = 0.011), whereas the difference between the ALK and KRAS mutation-positive groups was not significant (*p* > 0.05).

### Prediction of Survival Duration

**Figure 3:**
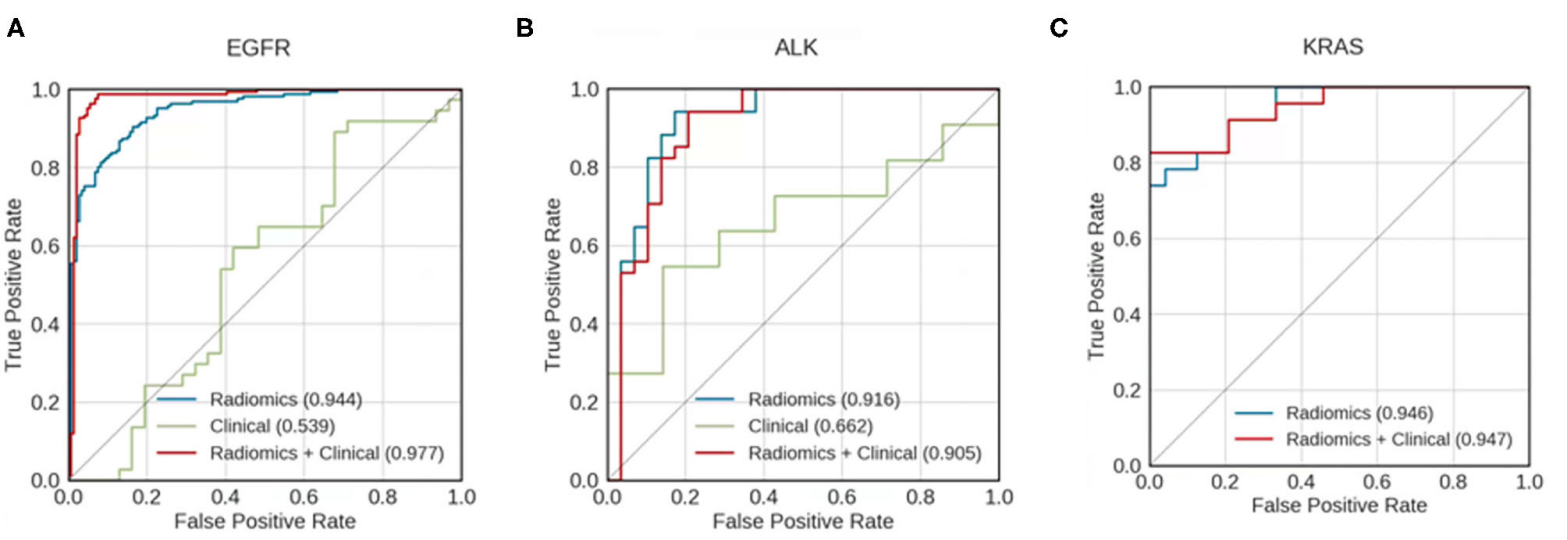
Receiver operating characteristic (ROC) curves for models predicting whether patients with mutations of **(A)** EGFR, **(B)** ALK, and **(C)** KRAS survived longer than the median survival duration of the mutation-positive group. Curves are shown for models using clinical data only (green), radiomics features only (blue), and a combination of both clinical data and radiomic features (red). The areas under the receiver operating characteristic curves (AUCs) are indicated in each panel. KRAS mutation—positive group has too small a sample size to build the predictive model using clinical data alone.

For all mutation-positive groups, the predictive performance of models built with radiomic features alone was better than that of models built with clinical data alone. Combining radiomic features and clinical data resulted in the most accurate prediction results (Figure 3). When using both clinical data and radiomic features in the modeling, the AUCs for predicting whether patients survived longer than the median survival duration of the group was 0.977, 0.905, and 0.947 for EGFR, ALK, and KRAS, respectively. Table 3 shows the accuracy, AUC, sensitivity, and specificity of the survival duration predictions for the patients in EGFR, ALK, or KRAS mutation-positive group, respectively. Both radiomic features and clinical data were combined to generate the performance data in Table 3. The accuracy was 94.9%, 84.1%, and 83.0% for the survival duration predictions for EGFR, ALK, and KRAS mutation-positive group, respectively. The sensitivity was 96.0, 88.0, and 83.0% for the survival duration predictions of EGFR, ALK, and KRAS mutation-positive group, respectively. The specificity was 94.0, 81.0, and 83.0% for the survival duration predictions of the patients in the EGFR, ALK, and KRAS mutation-positive groups, respectively.

**Table 3:** Performance metrics for predicting whether patients survive longer than the group median in EGFR, ALK, and KRAS mutation-positive groups.

**Mutation**	**Accuracy**	AUC^*^	**Sensitivity**	**Specificity**
EGFR	94.90%	0.977	96.00%	94.00%
ALK	84.10%	0.905	88.00%	81.00%
KRAS	83.00%	0.947	83.00%	83.00%

* *AUC, area under the receiver operating characteristic curve*.

*EGFR, epidermal growth factor receptor; ALK, anaplastic lymphoma kinase; KRAS, Kirsten rat sarcoma viral oncogene homolog*.

*Both clinical data and radiomics features were used for predictive modeling*.

### Cox Regression Analysis and Radiomic Score Calculation

**Figure 4:**
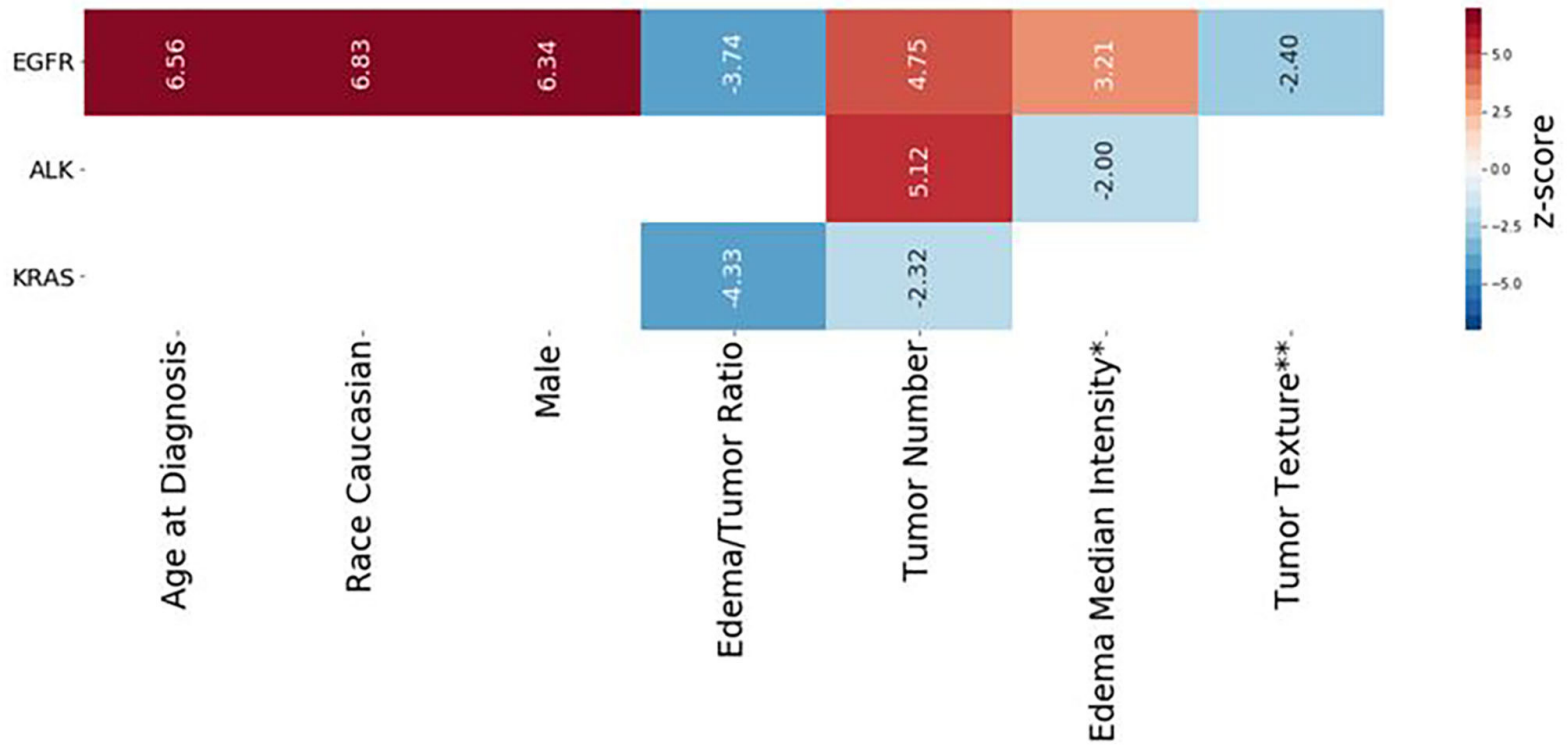
Radiomic scores of survival durations for EGFR, ALK, and KRAS mutation-positive groups. Each column represents the components of the radiomic score for survival prediction for each mutation-positive group, as indicated on the left end. The color indicates the z-score for each feature, based on multivariate Cox regression analysis, according to the scale shown on the right end. The numerical value of each Wald statistics is indicated with imbedded texts. Features with positive values (red) are associated with shorter survival duration, while those with negative values are associated with longer survival duration. The corresponding Cox regression coefficients of the features are shown in Table 4. *Edema Median Intensity: Edema_Intensity_squareroot_Intensity_Median. **Tumor Texture: Tumor Texture log-sigma-3-mm-3D GLRLM LongRunHighGrayLevelEmphasis.

Table 4 presents multivariate Cox regression results for the three mutation-positive groups. The demographic and radiomic features that were statistically significantly associated with survival duration (*p* < 0.05) are listed in Table 4. The features with positive coefficients were associated with shorter survival duration while those with negative coefficients were associated with longer survival duration. For the EGFR mutation-positive group, the radiomic score consisted of age {[Coefficient (coef): 2.76]}, Caucasian race (coef: 0.961), male sex (coef: 0.89), edema/tumor volume ratio (coef: −3.71), tumor number (coef: 1.78), an intensity feature exacted from edema area (coef: 1.37) and a textual feature exacted from tumor area (coef: −1.41). For the ALK mutation-positive group, the radiomic score consisted of the tumor number (coef: 3.05), and an intensity feature exacted from edema area (coef: −1.76). For the KRAS mutation-positive group, the radiomic score consisted of the edema/tumor volume ratio (coef: −16.8) and the tumor number (coef: −1.06). The feature names and the z score listed in Table 4 are graphically presented in Figure 4.

**Table 4:** Demographic and radiomic features significantly associated with survival duration for each mutation-positive group as determined by multivariate Cox regression analysis.

**Group**	**Features**	**coef**		**se(coef)**	***z***	***p***	**Lower 0.95**	**Upper 0.95**
EGFR	Age	2.76		0.42	6.56	<0.001	1.93	3.58
	Race Caucasian	0.96		0.14	6.83	<0.001	0.69	1.24
	Sex Male	0.89		0.14	6.34	<0.001	0.62	1.17
	Edema/Tumor Ratio	−3.71		0.99	−3.74	<0.001	−5.65	−1.76
	Tumor Number	1.78		0.38	4.75	<0.001	1.05	2.52
	Edema Median Intensity^*^	1.37		0.43	3.21	0.001	0.54	2.21
	Tumor Texture^**^	−1.41		0.59	−2.40	0.016	−2.56	−0.26
ALK	Tumor Number	3.05		0.60	5.12	<0.001	1.88	4.21
	Edema Median Intensity^*^	−1.76		0.88	−2.00	0.045	−3.48	−0.04
KRAS	Edema/Tumor Ratio	−16.80		3.89	−4.33	<0.001	−24.50	−9.22
	Tumor Number	−1.06		0.45	−2.32	0.020	−1.95	−0.17

Coef, Cox regression coefficient; se(coef), standard error of the Cox regression coefficient; lower 0.95, the lower bound of the 95% confidence interval; upper 0.95, the upper bound of the 95% confidence interval;

* Edema Median Intensity, Edema_Intensity_squareroot_Intensity_Median;

** *Tumor Texture, Tumor Texture log_sigma_3-mm_3D GLRLM LongRunHighGrayLevelEmphasis; EGFR, epidermal growth factor receptor; ALK, anaplastic lymphoma kinase; KRAS, Kirsten rat sarcoma viral oncogene homolog*.

**Figure 5:**
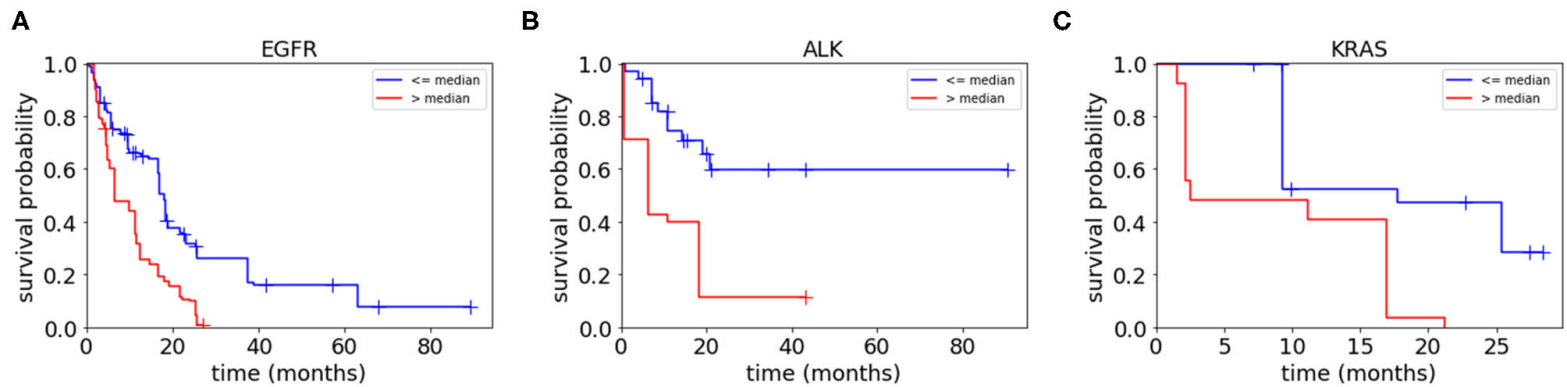
Kaplan–Meier plots for each mutation-positive group **(A–C)** separated into two subgroups by their radiomic scores (higher than or lower than the median radiomic score for each mutation-positive group). The subgroup with radiomic score values higher than the median radiomic score of each mutation-positive group had significantly shorter survival duration than the subgroup with values lower than the median radiomic score. The radiomic scores were computed as the weighted average of the features shown in Table 4 (weighted by Cox regression coefficients).

To assess the collective prognostic power of the features that were statistically significantly associated with the patients' survival, we constructed radiomic scores through a linear combination of the significant radiomic features listed in Table 4 which were weighted by the coefficients. We then divided each of the three patient groups into two subgroups based on the radiomic scores, i.e., assigning those patients with the radiomic scores lower than the median radiomic score of the group into a lower score subgroup and assigning the rest of the patients in the group into a higher score group. Figure 5 shows Kaplan–Meier plots of the two subgroups within each mutation-positive group based on radiomic scores. In each of the three mutation-positive groups, the subgroup with lower radiomic score had longer median survival duration than that of the subgroup with higher radiomic score.

## Discussion

In this study, we built machine learning models to predict whether patients with EGFR, ALK, or KRAS mutation-positive primary NSCLC survived longer than the median survival duration for each specific mutation group. The final models of our study used 50 radiomic features together with 18 clinical features and achieved AUC of 0.977, 0.905, and 0.947 for the three mutation-positive groups, i.e., EGFR, ALK, and KRAS groups, respectively. Subsequently, we identified radiomic and clinical features significantly associated with survival duration for the patients in the three mutation-positive groups. Finally, we constructed radiomic scores using linear combinations of these features weighted with their coefficients in the multivariate regression. After dividing each of the three mutation groups into two subgroups according to radiomic scores, our study showed that the subgroup with lower radiomic scores had statistically significant longer median survival duration, indicating strong association between radiomic scores and the patients' survival duration.

The performance of our predictive models compared favorably to those of published predictive models based on the computed tomography (CT) images of primary lung cancer (25–28). Hosny et al. (29) used a 3D convolutional neural network (CNN) to study prognostic stratification in a multi-cohort radiomic study using the lung CT images of 1,194 patients with NSCLC. Their models predicted whether patients could survive longer than 2 years after treated either with radiotherapy or surgery, and achieved AUC of 0.70 and 0.71, respectively. It is challenging to compare our results, which were based on the MRI radiomics of brain metastases, to the results of the deep learning study which was based on lung CT images. Nevertheless, judging by AUC values alone, the performance of our predictive models was comparable to the work performed by deep learning networks (29).

Our predictive models achieved reasonable performance as compared to other studies using radiomic features from MR images of brain metastases (30–33). For example, Béresová et al. (33) demonstrated that using MR image-based textural radiomic analysis could distinguish brain metastases originating from lung cancer vs. breast cancer, achieving AUC of 0.70. In another study, Ortiz-Ramon et al. (32) used radiomic features extracted from MR images of brain metastases to predict whether the primary cancer being lung cancer or melanoma, achieving AUC of 0.95. Recently, Kniep et al., build predictive models using radiomic features from MR images to predict whether brain metastases originated from primary breast cancer, small cell lung cancer, NSCLC, gastrointestinal cancer, or melanoma. The AUC of their predictive models were between 0.64 for NSCLC and 0.82 for breast cancer (34).

Our approach using radiomic scores to predict survival duration of NSCLC patients with brain metastases was novel. We constructed radiomic scores with linear combinations of 2–7 significant radiomic features for each mutation-positive group, weighted by their Cox coefficients. Our radiomic score calculations indicated that different sets of radiomic features were significantly associated with survival duration in different mutation groups. For example, an edema feature, the Edema_Intensity_squareroot_Intensity_Median, was significantly associated with survival duration of patients in the EGFR and ALK mutation-positive groups, but not in the KRAS mutation-positive group. Edema Tumor Volume ratio on the other hand, was significantly associated with survival duration in the EGFR and KRAS mutation-positive groups, but not in ALK mutation-positive group. Our findings indicated the potential mutation-specific association between the radiomic features and survival durations. These results were not unexpected since our radiomic scores were consisted of features reflecting tumor heterogeneity such as edema intensity and tumor texture which have been known to affect survival (35).

Our findings regarding the relationship between peritumoral edema of brain metastases and the survival durations is generally in line with published literature (35, 36). Spanberger et al. studied the prognostic value of the extent of peritumoral brain edema in the patients operated for single brain metastasis. They reported a strong correlation between the extent of peritumoral edema on brain MRI scans and overall survival, i.e., patients with small peritumoral edema have longer survival than patients with large peritumoral edema (35). Our current study showed similar findings, i.e., lower edema/tumor ratio in our radiomic scores indicated longer survival duration. In addition, Berghoff et al. studied the role of tumor-infiltrating lymphocytes (TIFs) in the immune microenvironment of 116 specimen of brain metastases originating from different primary cancers including lung cancer, breast cancer, melanoma, and renal cell carcinoma. They found that dense TIFs correlated with peritumoral brain edema and the overall survival (36). A recent study by Nardone et al. (37) has also shown that the peritumoral edema and tumor volume of brain metastases were correlated with overall survival in patients with NSCLC undergoing radiosurgery. Taken together of the prior published reports and our current study, there is supporting evidence for incorporating brain tumor characteristics such as edema and tumor volume into survival analysis of patients with brain metastases.

The multivariate Cox regression in our study showed that age at diagnosis, Caucasian race, and male gender, were highly correlated with survival duration in the EGFR mutation-positive group. This result was consistent with literature indicating that age, active extracranial disease, and EGFR mutation are independently associated with survival (9). However, it is challenging to compare our analysis of survival duration with others because of differences in study cohorts, systemic disease status and treatment regimen for both the primary cancers and brain metastases. Nevertheless, it is reasonable to evaluate survival in terms of mutation status since molecular targeted therapy based on mutation information may improve prognosis and survival (38). For instance, the progression-free and overall survival of patients with EGFR and ALK mutations may be improved by treatment with tyrosine kinase inhibitors and ALK inhibitors specifically targeting these two mutations (38). Our study results provide the pilot data supporting radiomic scores as non-invasive biomarkers for assessment of survival duration in lung cancer brain metastases according to the mutation status. Nevertheless, independent validation is needed to substantiate our results.

There were several limitations to this study. First, this was a retrospective study focusing on NSCLC patients with brain metastases who were treated at a single institution over a 9-year interval. Our study design was inherently limited by various confounding variables, such as patient characteristics, imaging parameters, and treatment regimens for the primary NSCLC. Second, our sample size was modest, which might have limited our ability to build more robust predictive models with radiomic features. Third, the mutation status for this cohort was obtained from the primary NSCLC. Since most patients in our cohort did not undergo invasive biopsy or surgery of the brain metastases, the brain metastases could not be directly genotyped and we therefore assumed that brain metastases having the same mutation status as the primary NSCLC. We recognize this limitation with the understanding that mutation status in the primary NSCLC and distant metastases may not always be concordant (39). Lastly, this pilot study did not evaluate or control for all the potential confounding factors that might have contributed to survival duration, such as primary tumor status, systematic disease status, neurological deficits, and treatment regimen for the primary NSCLC and brain metastases. This was because we did not have the statistical power in this retrospective study with a modest sample size to control for all the highly variable confounding factors affecting survival. We recognize our approach for building predictive models with the potential uncontrolled variables may have affected our model performance. We will consider those confounding factors in our future large-scale multicenter research.

Despite these limitations, our study had strengths. First, to the best of our knowledge, our study was the first to use MRI radiomics of brain metastases and machine learning algorithms to predict the survival durations of patients with NSCLC, accounting for their mutation status. Second, we used a 3D slice-by-slice approach to segment brain metastases in their entirety, which we believe should have provided a more detailed characterization of tumor heterogeneity than what could be achieved using a 2D method (32). Third, we constructed radiomic scores using both radiomic features and clinical data, which improved predictive power compared to the scores constructed using either clinical data or radiomic data alone. Therefore, our study has merit as an exploratory, proof-of-concept pilot study from which to generate hypotheses for future large-scale, multicenter studies using imaging biomarkers to predict survival durations of patients with brain metastases from NSCLC and other primary cancers.

In summary, our study showed that a MRI radiomic approach capturing the critical radiological features of brain metastases in patients with primary NSCLC may be used to predict survival durations according to mutation status. Our data supports the concept of using radiomic scores as non-invasive imaging biomarkers for survival analysis, which is important for personalized treatment and prognostic assessment for cancer patients with metastatic disease.

## Data Availability Statement

The raw data supporting the conclusions of this article will be made available by the authors to qualified researchers, without undue reservation.

## Ethics Statement

The studies involving human participants were reviewed and approved by the Institutional Review Board at City of Hope National Medical Center which approved this study and waived informed consent due to its retrospective nature. Written informed consent for participation was not required for this study in accordance with the institutional requirements.

## Author Contributions

BC and RS designed and conducted the study. NY, TJ, IM, TW, BC, and RS analyzed the brain MR imaging data. NY, TW, and BC performed tumor segmentation and reviewed the segmented images for consistency. TJ developed the pipeline for predictive modeling and machine learning. TJ and NY performed statistical analysis. BC, TJ, NY, IM, CW, TW, ZC, RR, RC, AH, SS, and RS contributed to data interpretation. BC, TJ, NY, IM, and RS contributed to the manuscript writing process and BTC prepared the first draft of the entire manuscript. All authors approved the final manuscript.

## Conflict of Interest

The authors declare that the research was conducted in the absence of any commercial or financial relationships that could be construed as a potential conflict of interest.
