# Supplementary material for: Predicting Survival Duration With MRI Radiomics of Brain Metastases From Non-small Cell Lung Cancer
Source: Front Oncol. 2021 Mar 5;11:621088. doi: 10.3389/fonc.2021.621088 (PMC7973105; doi:10.3389/fonc.2021.621088)
Supplement: Supplementary file 1 [file Table_1.pdf]

Supplementary Material

Table 1S. Top 20 features for predicting whether patients survive longer than the group median in EGFR, ALK, and KRAS mutation-positive groups.

Mutation	Region	Feature type	Filter #	Feature group##	Feature Name
EGFR	Edema	Intensity	squareroot	Intensity	Median*
			logarithm	Intensity	Kurtosis
		Texture	original	GLCM	ClusterShade
			logarithm	GLSZM	GrayLevelNonUniformity
			logarithm	NGTDM	Contrast
			original	GLDM	LargeDependenceLowGrayLevelEmphasis
			logarithm	GLRLM	LongRunLowGrayLevelEmphasis
			square	GLDM	LowGrayLevelEmphasis
			wavelet-LH	NGTDM	Coarseness
	Tumor	Intensity	logarithm	Intensity	Maximum
			lbp-2D	Intensity	MeanAbsoluteDeviation
			exponential	Intensity	RobustMeanAbsoluteDeviation
		Texture	log-sigma-3-mm-3D	GLRLM	LongRunHighGrayLevelEmphasis**
			logarithm	NGTDM	Busyness
			log-sigma-1-mm-3D	NGTDM	Busyness
			gradient	GLSZM	SmallAreaLowGrayLevelEmphasis
			squareroot	GLCM	Contrast
			original	GLRLM	RunVariance
			gradient	GLCM	Imc1
			squareroot	NGTDM	Coarseness
ALK	Edema	Intensity	log-sigma-3-mm-3D	Intensity	Skewness
			squareroot	Intensity	Median*
			logarithm	Intensity	Uniformity
			logarithm	Intensity	Median
		Texture	logarithm	GLRLM	ShortRunEmphasis
			wavelet-HL	GLRLM	RunEntropy
			wavelet-HL	GLRLM	RunPercentage
			log-sigma-1-mm-3D	GLDM	LargeDependenceHighGrayLevelEmphasis
			wavelet-HL	GLRLM	RunLengthNonUniformityNormalized

			squareroot	NGTDM	Coarseness
			wavelet-HH	GLSZM	ZoneEntropy
			original	GLDM	SmallDependenceEmphasis
			log-sigma-2-mm-3D	GLRLM	HighGrayLevelRunEmphasis
			wavelet-LL	GLDM	LargeDependenceLowGrayLevelEmphasis
	Tumor	Intensity	square	Intensity	RootMeanSquared
		Texture	log-sigma-2-mm-3D	GLDM	LargeDependenceEmphasis
			logarithm	GLCM	JointEntropy
			square	NGTDM	Contrast
			square	GLCM	SumAverage
			logarithm	GLCM	Id
KRAS	Edema	Intensity	wavelet-LH	Intensity	RootMeanSquared
			logarithm	Intensity	Median
			logarithm	Intensity	Mean
			logarithm	Intensity	10Percentile
			logarithm	Intensity	90Percentile
		Texture	wavelet-HL	GLRLM	RunEntropy
			logarithm	GLCM	SumEntropy
			wavelet-LH	GLSZM	GrayLevelNonUniformityNormalized
			log-sigma-1-mm-3D	GLSZM	SmallAreaLowGrayLevelEmphasis
			exponential	GLSZM	SizeZoneNonUniformityNormalized
			wavelet-HL	GLRLM	RunPercentage
			square	GLSZM	HighGrayLevelZoneEmphasis
	Tumor	Texture	exponential	GLSZM	SmallAreaLowGrayLevelEmphasis
			original	shape	Flatness
			wavelet-LH	GLCM	Imc2
			exponential	NGTDM	Strength
			log-sigma-1-mm-3D	GLRLM	RunPercentage
			gradient	NGTDM	Busyness
			log-sigma-1-mm-3D	GLDM	DependenceNonUniformityNormalized
			gradient	NGTDM	Contrast

[#]Notes on filters: *log-sigma-n-mm-3D*—a Laplacian of Gaussian image, Gaussian kernel set to be n (=1, 2, 3) mm, three-dimensional data was used. *wavelet-**—2D wavelet transform was used, the image was decomposed into two axes. LH (low-high) means this filter focuses on y-axis variation, showing the horizontal details of the image. HH (high-high) means this filter focuses on both x-axis and y-axis variations, showing the diagonal details of the image. *lbp-2D*—local binary pattern (lbp) in 2D.

[##]Feature group abbreviations: *GLCM,* Gray Level Co-occurrence Matrix; *GLRLM,* Gray Level Run Length Matrix; *GLSZM,* Gray Level Size Zone Matrix; *NGTDM,* Neighboring Gray Tone Difference Matrix; *GLDM,* Gray Level Dependence Matrix.

*The edema intensity feature shown in Fig. 4 and Table 4.

**The tumor texture feature shown in Fig. 4 and Table 4.
